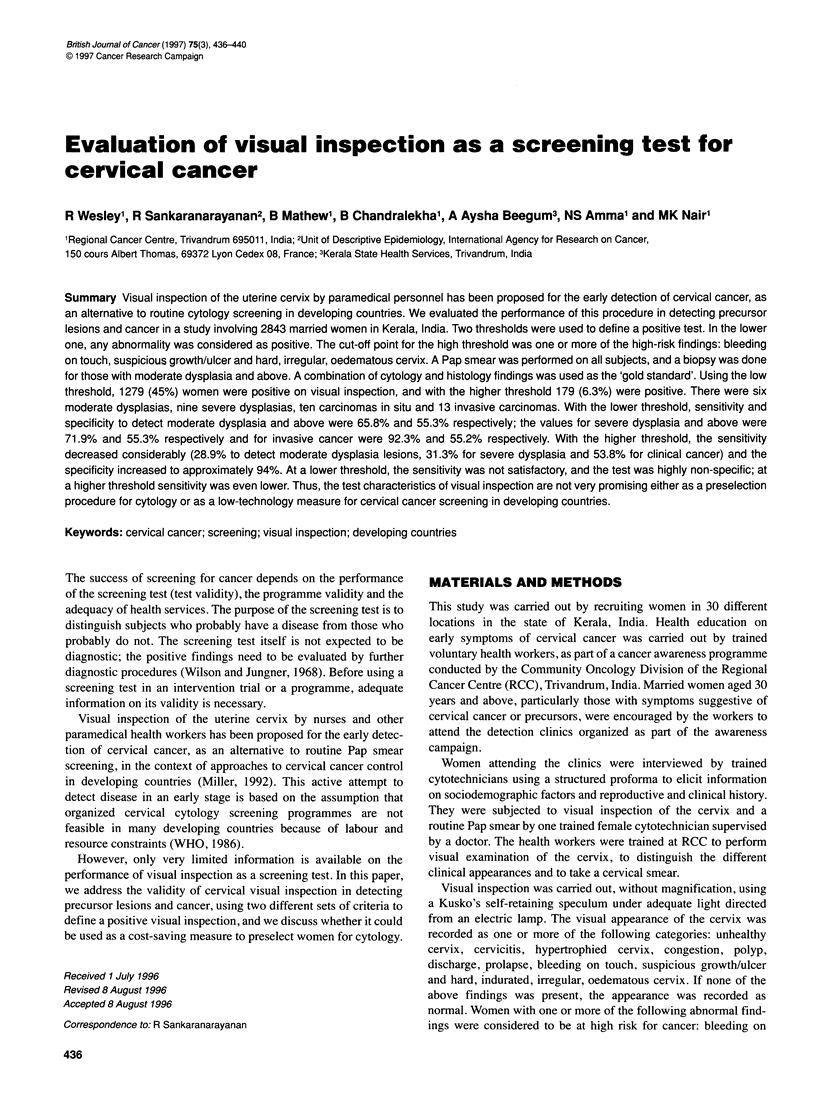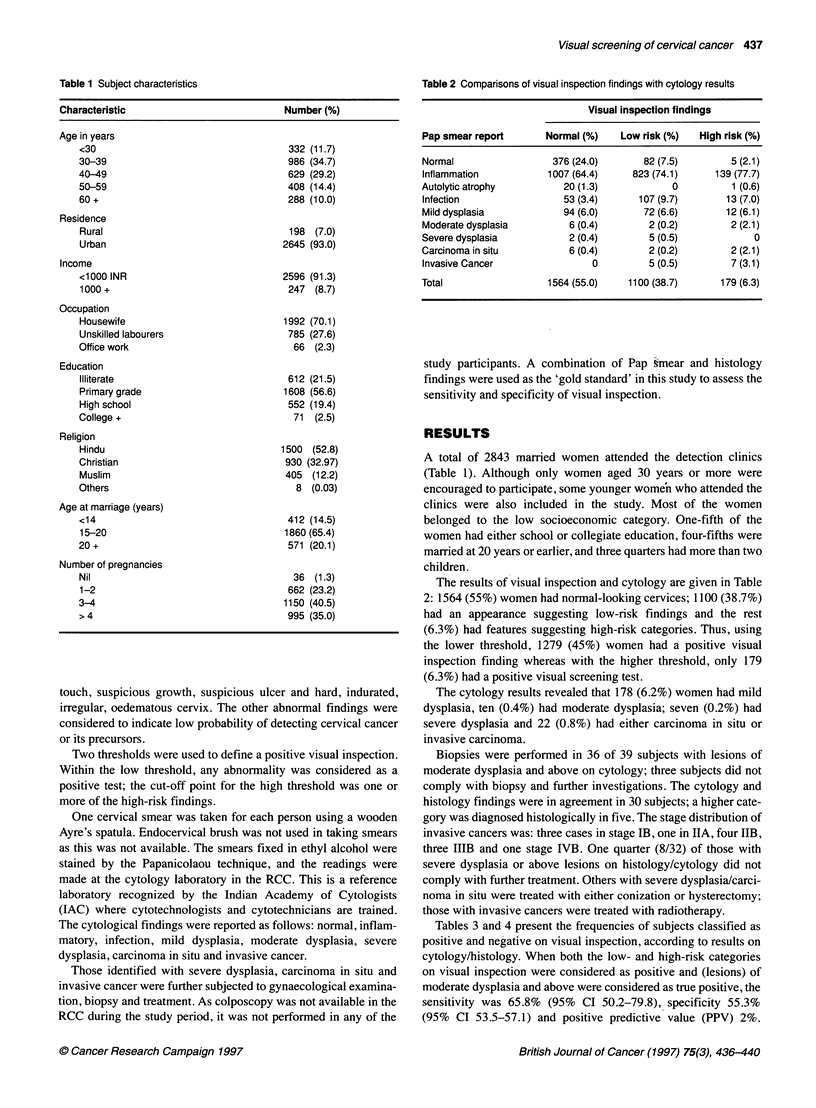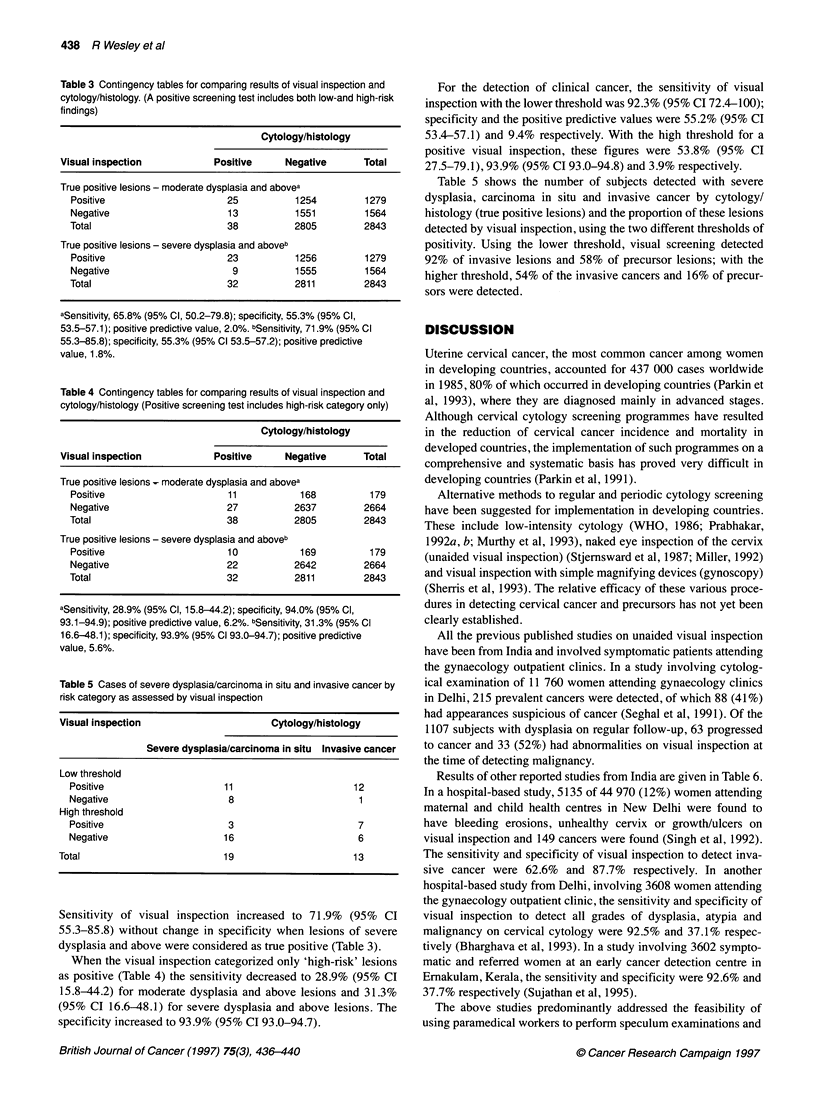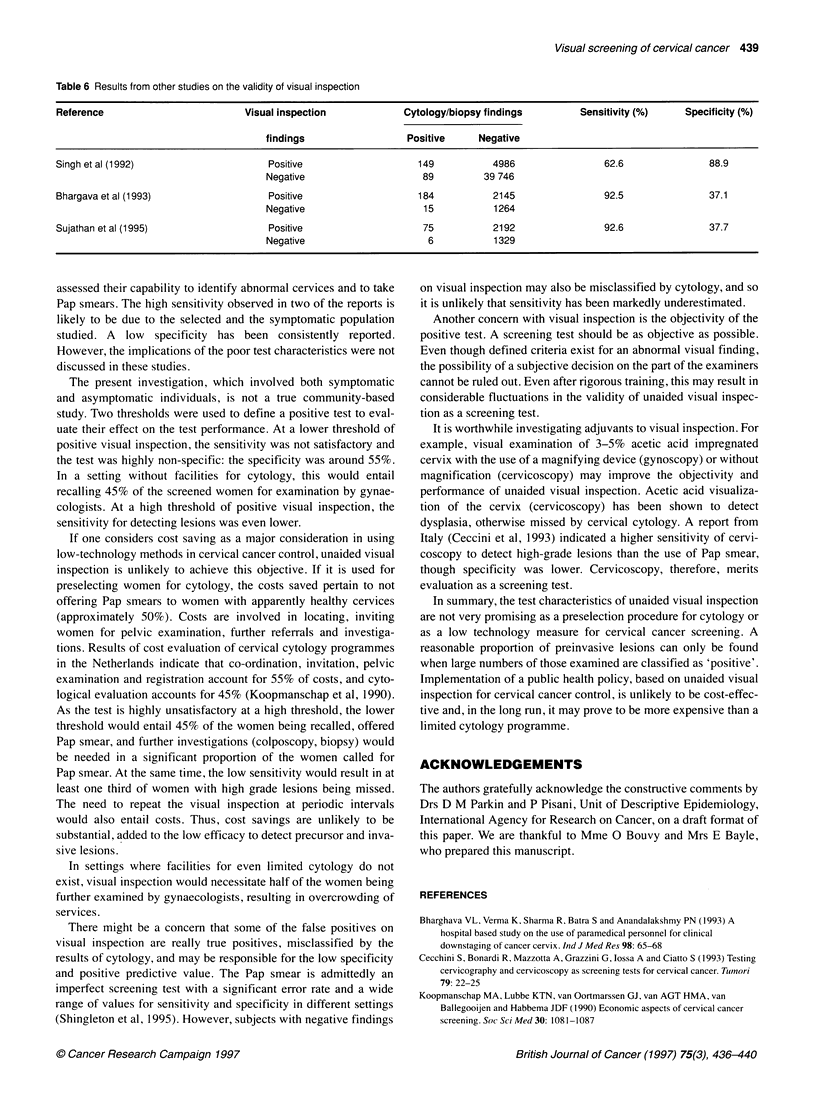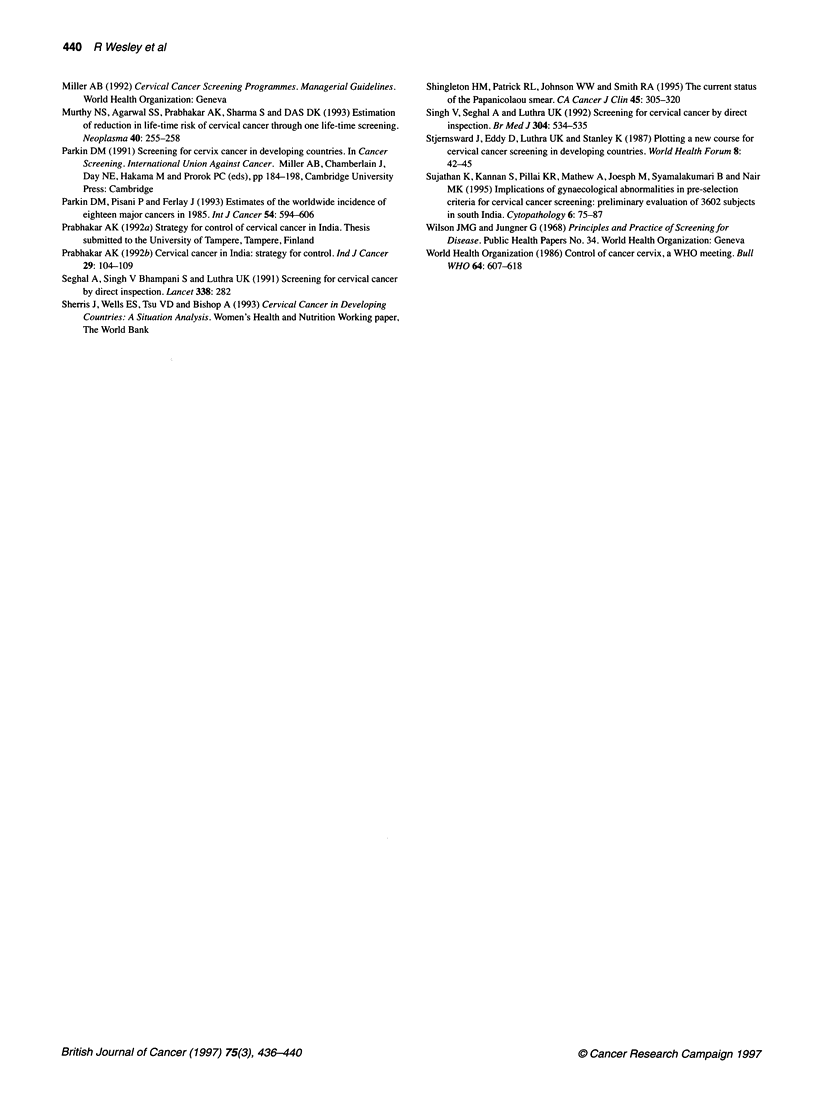# Evaluation of visual inspection as a screening test for cervical cancer.

**DOI:** 10.1038/bjc.1997.72

**Published:** 1997

**Authors:** R. Wesley, R. Sankaranarayanan, B. Mathew, B. Chandralekha, A. Aysha Beegum, N. S. Amma, M. K. Nair

**Affiliations:** Regional Cancer Centre, Trivandrum, India.

## Abstract

Visual inspection of the uterine cervix by paramedical personnel has been proposed for the early detection of cervical cancer, as an alternative to routine cytology screening in developing countries. We evaluated the performance of this procedure in detecting precursor lesions and cancer in a study involving 2843 married women in Kerala, India. Two thresholds were used to define a positive test. In the lower one, any abnormality was considered as positive. The cut-off point for the high threshold was one or more of the high-risk findings: bleeding on touch, suspicious growth/ulcer and hard, irregular, oedematous cervix. A Pap smear was performed on all subjects, and a biopsy was done for those with moderate dysplasia and above. A combination of cytology and histology findings was used as the 'gold standard'. Using the low threshold, 1279 (45%) women were positive on visual inspection, and with the higher threshold 179 (6.3%) were positive. There were six moderate dysplasias, nine severe dysplasias, ten carcinomas in situ and 13 invasive carcinomas. With the lower threshold, sensitivity and specificity to detect moderate dysplasia and above were 65.8% and 55.3% respectively; the values for severe dysplasia and above were 71.9% and 55.3% respectively and for invasive cancer were 92.3% and 55.2% respectively. With the higher threshold, the sensitivity decreased considerably (28.9% to detect moderate dysplasia lesions, 31.3% for severe dysplasia and 53.8% for clinical cancer) and the specificity increased to approximately 94%. At a lower threshold, the sensitivity was not satisfactory, and the test was highly non-specific; at a higher threshold sensitivity was even lower. Thus, the test characteristics of visual inspection are not very promising either as a preselection procedure for cytology or as a low-technology measure for cervical cancer screening in developing countries.


British Journal of Cancer (1997) 75(3), 436–440
© 1997 Cancer Research Campaign

### Evaluation of visual inspection as a screening test for cervical cancer

**R Wesley[1], R Sankaranarayanan[2], B Mathew[1], B Chandralekha[1], A Aysha Beegum[3], NS Amma[1] and MK Nair[1]**

[1]Regional Cancer Centre, Trivandrum 695011, India; [2]Unit of Descriptive Epidemiology, International Agency for Research on Cancer, 150 cours Albert Thomas, 69372 Lyon Cedex 08, France; [3]Kerala State Health Services, Trivandrum, India

**Summary** Visual inspection of the uterine cervix by paramedical personnel has been proposed for the early detection of cervical cancer, as an alternative to routine cytology screening in developing countries. We evaluated the performance of this procedure in detecting precursor lesions and cancer in a study involving 2843 married women in Kerala, India. Two thresholds were used to define a positive test. In the lower one, any abnormality was considered as positive. The cut-off point for the high threshold was one or more of the high-risk findings: bleeding on touch, suspicious growth/ulcer and hard, irregular, oedematous cervix. A Pap smear was performed on all subjects, and a biopsy was done for those with moderate dysplasia and above. A combination of cytology and histology findings was used as the 'gold standard'. Using the low threshold, 1279 (45%) women were positive on visual inspection, and with the higher threshold 179 (6.3%) were positive. There were six moderate dysplasias, nine severe dysplasias, ten carcinomas in situ and 13 invasive carcinomas. With the lower threshold, sensitivity and specificity to detect moderate dysplasia and above were 65.8% and 55.3% respectively; the values for severe dysplasia and above were 71.9% and 55.3% respectively and for invasive cancer were 92.3% and 55.2% respectively. With the higher threshold, the sensitivity decreased considerably (28.9% to detect moderate dysplasia lesions, 31.3% for severe dysplasia and 53.8% for clinical cancer) and the specificity increased to approximately 94%. At a lower threshold, the sensitivity was not satisfactory, and the test was highly non-specific; at a higher threshold sensitivity was even lower. Thus, the test characteristics of visual inspection are not very promising either as a preselection procedure for cytology or as a low-technology measure for cervical cancer screening in developing countries.

**Keywords:** cervical cancer; screening; visual inspection; developing countries

The success of screening for cancer depends on the performance of the screening test (test validity), the programme validity and the adequacy of health services. The purpose of the screening test is to distinguish subjects who probably have a disease from those who probably do not. The screening test itself is not expected to be diagnostic; the positive findings need to be evaluated by further diagnostic procedures (Wilson and Jungner, 1968). Before using a screening test in an intervention trial or a programme, adequate information on its validity is necessary.

Visual inspection of the uterine cervix by nurses and other paramedical health workers has been proposed for the early detection of cervical cancer, as an alternative to routine Pap smear screening, in the context of approaches to cervical cancer control in developing countries (Miller, 1992). This active attempt to detect disease in an early stage is based on the assumption that organized cervical cytology screening programmes are not feasible in many developing countries because of labour and resource constraints (WHO, 1986).

However, only very limited information is available on the performance of visual inspection as a screening test. In this paper, we address the validity of cervical visual inspection in detecting precursor lesions and cancer, using two different sets of criteria to define a positive visual inspection, and we discuss whether it could be used as a cost-saving measure to preselect women for cytology.

*Received 1 July 1996*
*Revised 8 August 1996*
*Accepted 8 August 1996*

*Correspondence to:* R Sankaranarayanan

### MATERIALS AND METHODS

This study was carried out by recruiting women in 30 different locations in the state of Kerala, India. Health education on early symptoms of cervical cancer was carried out by trained voluntary health workers, as part of a cancer awareness programme conducted by the Community Oncology Division of the Regional Cancer Centre (RCC), Trivandrum, India. Married women aged 30 years and above, particularly those with symptoms suggestive of cervical cancer or precursors, were encouraged by the workers to attend the detection clinics organized as part of the awareness campaign.

Women attending the clinics were interviewed by trained cytotechnicians using a structured proforma to elicit information on sociodemographic factors and reproductive and clinical history. They were subjected to visual inspection of the cervix and a routine Pap smear by one trained female cytotechnician supervised by a doctor. The health workers were trained at RCC to perform visual examination of the cervix, to distinguish the different clinical appearances and to take a cervical smear.

Visual inspection was carried out, without magnification, using a Kusko's self-retaining speculum under adequate light directed from an electric lamp. The visual appearance of the cervix was recorded as one or more of the following categories: unhealthy cervix, cervicitis, hypertrophied cervix, congestion, polyp, discharge, prolapse, bleeding on touch, suspicious growth/ulcer and hard, indurated, irregular, oedematous cervix. If none of the above findings was present, the appearance was recorded as normal. Women with one or more of the following abnormal findings were considered to be at high risk for cancer: bleeding on

**Table 1** Subject characteristics

| Characteristic | Number (%) |
|---|---|
| Age in years | |
| <30 | 332 (11.7) |
| 30–39 | 986 (34.7) |
| 40–49 | 629 (29.2) |
| 50–59 | 408 (14.4) |
| 60 + | 288 (10.0) |
| Residence | |
| Rural | 198 (7.0) |
| Urban | 2645 (93.0) |
| Income | |
| <1000 INR | 2596 (91.3) |
| 1000 + | 247 (8.7) |
| Occupation | |
| Housewife | 1992 (70.1) |
| Unskilled labourers | 785 (27.6) |
| Office work | 66 (2.3) |
| Education | |
| Illiterate | 612 (21.5) |
| Primary grade | 1608 (56.6) |
| High school | 552 (19.4) |
| College + | 71 (2.5) |
| Religion | |
| Hindu | 1500 (52.8) |
| Christian | 930 (32.97) |
| Muslim | 405 (12.2) |
| Others | 8 (0.03) |
| Age at marriage (years) | |
| <14 | 412 (14.5) |
| 15–20 | 1860 (65.4) |
| 20 + | 571 (20.1) |
| Number of pregnancies | |
| Nil | 36 (1.3) |
| 1–2 | 662 (23.2) |
| 3–4 | 1150 (40.5) |
| > 4 | 995 (35.0) |

touch, suspicious growth, suspicious ulcer and hard, indurated, irregular, oedematous cervix. The other abnormal findings were considered to indicate low probability of detecting cervical cancer or its precursors.

Two thresholds were used to define a positive visual inspection. Within the low threshold, any abnormality was considered as a positive test; the cut-off point for the high threshold was one or more of the high-risk findings.

One cervical smear was taken for each person using a wooden Ayre's spatula. Endocervical brush was not used in taking smears as this was not available. The smears fixed in ethyl alcohol were stained by the Papanicolaou technique, and the readings were made at the cytology laboratory in the RCC. This is a reference laboratory recognized by the Indian Academy of Cytologists (IAC) where cytotechnologists and cytotechnicians are trained. The cytological findings were reported as follows: normal, inflammatory, infection, mild dysplasia, moderate dysplasia, severe dysplasia, carcinoma in situ and invasive cancer.

Those identified with severe dysplasia, carcinoma in situ and invasive cancer were further subjected to gynaecological examination, biopsy and treatment. As colposcopy was not available in the RCC during the study period, it was not performed in any of the study participants. A combination of Pap smear and histology findings were used as the 'gold standard' in this study to assess the sensitivity and specificity of visual inspection.

**Table 2** Comparisons of visual inspection findings with cytology results

| | Visual inspection findings | | |
|---|---|---|---|
| Pap smear report | Normal (%) | Low risk (%) | High risk (%) |
| Normal | 376 (24.0) | 82 (7.5) | 5 (2.1) |
| Inflammation | 1007 (64.4) | 823 (74.1) | 139 (77.7) |
| Autolytic atrophy | 20 (1.3) | 0 | 1 (0.6) |
| Infection | 53 (3.4) | 107 (9.7) | 13 (7.0) |
| Mild dysplasia | 94 (6.0) | 72 (6.6) | 12 (6.1) |
| Moderate dysplasia | 6 (0.4) | 2 (0.2) | 2 (2.1) |
| Severe dysplasia | 2 (0.4) | 5 (0.5) | 0 |
| Carcinoma in situ | 6 (0.4) | 2 (0.2) | 2 (2.1) |
| Invasive Cancer | 0 | 5 (0.5) | 7 (3.1) |
| Total | 1564 (55.0) | 1100 (38.7) | 179 (6.3) |

### RESULTS

A total of 2843 married women attended the detection clinics (Table 1). Although only women aged 30 years or more were encouraged to participate, some younger women who attended the clinics were also included in the study. Most of the women belonged to the low socioeconomic category. One-fifth of the women had either school or collegiate education, four-fifths were married at 20 years or earlier, and three quarters had more than two children.

The results of visual inspection and cytology are given in Table 2: 1564 (55%) women had normal-looking cervices; 1100 (38.7%) had an appearance suggesting low-risk findings and the rest (6.3%) had features suggesting high-risk categories. Thus, using the lower threshold, 1279 (45%) women had a positive visual inspection finding whereas with the higher threshold, only 179 (6.3%) had a positive visual screening test.

The cytology results revealed that 178 (6.2%) women had mild dysplasia, ten (0.4%) had moderate dysplasia; seven (0.2%) had severe dysplasia and 22 (0.8%) had either carcinoma in situ or invasive carcinoma.

Biopsies were performed in 36 of 39 subjects with lesions of moderate dysplasia and above on cytology; three subjects did not comply with biopsy and further investigations. The cytology and histology findings were in agreement in 30 subjects; a higher category was diagnosed histologically in five. The stage distribution of invasive cancers was: three cases in stage IB, one in IIA, four IIB, three IIIB and one stage IVB. One quarter (8/32) of those with severe dysplasia or above lesions on histology/cytology did not comply with further treatment. Others with severe dysplasia/carcinoma in situ were treated with either conization or hysterectomy; those with invasive cancers were treated with radiotherapy.

Tables 3 and 4 present the frequencies of subjects classified as positive and negative on visual inspection, according to results on cytology/histology. When both the low- and high-risk categories on visual inspection were considered as positive and (lesions) of moderate dysplasia and above were considered as true positive, the sensitivity was 65.8% (95% CI 50.2–79.8), specificity 55.3% (95% CI 53.5–57.1) and positive predictive value (PPV) 2%.

© Cancer Research Campaign 1997

**Table 3** Contingency tables for comparing results of visual inspection and cytology/histology. (A positive screening test includes both low-and high-risk findings)

| Visual inspection | Cytology/histology Positive | Negative | Total |
|---|---|---|---|
| True positive lesions – moderate dysplasia and above[a] | | | |
| Positive | 25 | 1254 | 1279 |
| Negative | 13 | 1551 | 1564 |
| Total | 38 | 2805 | 2843 |
| True positive lesions – severe dysplasia and above[b] | | | |
| Positive | 23 | 1256 | 1279 |
| Negative | 9 | 1555 | 1564 |
| Total | 32 | 2811 | 2843 |

[a]Sensitivity, 65.8% (95% CI, 50.2–79.8); specificity, 55.3% (95% CI, 53.5–57.1); positive predictive value, 2.0%. [b]Sensitivity, 71.9% (95% CI 55.3–85.8); specificity, 55.3% (95% CI 53.5–57.2); positive predictive value, 1.8%.

**Table 4** Contingency tables for comparing results of visual inspection and cytology/histology (Positive screening test includes high-risk category only)

| Visual inspection | Cytology/histology Positive | Negative | Total |
|---|---|---|---|
| True positive lesions – moderate dysplasia and above[a] | | | |
| Positive | 11 | 168 | 179 |
| Negative | 27 | 2637 | 2664 |
| Total | 38 | 2805 | 2843 |
| True positive lesions – severe dysplasia and above[b] | | | |
| Positive | 10 | 169 | 179 |
| Negative | 22 | 2642 | 2664 |
| Total | 32 | 2811 | 2843 |

[a]Sensitivity, 28.9% (95% CI, 15.8–44.2); specificity, 94.0% (95% CI, 93.1–94.9); positive predictive value, 6.2%. [b]Sensitivity, 31.3% (95% CI 16.6–48.1); specificity, 93.9% (95% CI 93.0–94.7); positive predictive value, 5.6%.

**Table 5** Cases of severe dysplasia/carcinoma in situ and invasive cancer by risk category as assessed by visual inspection

| Visual inspection | Cytology/histology Severe dysplasia/carcinoma in situ | Invasive cancer |
|---|---|---|
| Low threshold | | |
| Positive | 11 | 12 |
| Negative | 8 | 1 |
| High threshold | | |
| Positive | 3 | 7 |
| Negative | 16 | 6 |
| Total | 19 | 13 |

Sensitivity of visual inspection increased to 71.9% (95% CI 55.3–85.8) without change in specificity when lesions of severe dysplasia and above were considered as true positive (Table 3).

When the visual inspection categorized only 'high-risk' lesions as positive (Table 4) the sensitivity decreased to 28.9% (95% CI 15.8–44.2) for moderate dysplasia and above lesions and 31.3% (95% CI 16.6–48.1) for severe dysplasia and above lesions. The specificity increased to 93.9% (95% CI 93.0–94.7).

For the detection of clinical cancer, the sensitivity of visual inspection with the lower threshold was 92.3% (95% CI 72.4–100); specificity and the positive predictive values were 55.2% (95% CI 53.4–57.1) and 9.4% respectively. With the high threshold for a positive visual inspection, these figures were 53.8% (95% CI 27.5–79.1), 93.9% (95% CI 93.0–94.8) and 3.9% respectively.

Table 5 shows the number of subjects detected with severe dysplasia, carcinoma in situ and invasive cancer by cytology/histology (true positive lesions) and the proportion of these lesions detected by visual inspection, using the two different thresholds of positivity. Using the lower threshold, visual screening detected 92% of invasive lesions and 58% of precursor lesions; with the higher threshold, 54% of the invasive cancers and 16% of precursors were detected.

### DISCUSSION

Uterine cervical cancer, the most common cancer among women in developing countries, accounted for 437 000 cases worldwide in 1985, 80% of which occurred in developing countries (Parkin et al, 1993), where they are diagnosed mainly in advanced stages. Although cervical cytology screening programmes have resulted in the reduction of cervical cancer incidence and mortality in developed countries, the implementation of such programmes on a comprehensive and systematic basis has proved very difficult in developing countries (Parkin et al, 1991).

Alternative methods to regular and periodic cytology screening have been suggested for implementation in developing countries. These include low-intensity cytology (WHO, 1986; Prabhakar, 1992*a*, *b*; Murthy et al, 1993), naked eye inspection of the cervix (unaided visual inspection) (Stjernsward et al, 1987; Miller, 1992) and visual inspection with simple magnifying devices (gynoscopy) (Sherris et al, 1993). The relative efficacy of these various procedures in detecting cervical cancer and precursors has not yet been clearly established.

All the previous published studies on unaided visual inspection have been from India and involved symptomatic patients attending the gynaecology outpatient clinics. In a study involving cytological examination of 11 760 women attending gynaecology clinics in Delhi, 215 prevalent cancers were detected, of which 88 (41%) had appearances suspicious of cancer (Seghal et al, 1991). Of the 1107 subjects with dysplasia on regular follow-up, 63 progressed to cancer and 33 (52%) had abnormalities on visual inspection at the time of detecting malignancy.

Results of other reported studies from India are given in Table 6. In a hospital-based study, 5135 of 44 970 (12%) women attending maternal and child health centres in New Delhi were found to have bleeding erosions, unhealthy cervix or growth/ulcers on visual inspection and 149 cancers were found (Singh et al, 1992). The sensitivity and specificity of visual inspection to detect invasive cancer were 62.6% and 87.7% respectively. In another hospital-based study from Delhi, involving 3608 women attending the gynaecology outpatient clinic, the sensitivity and specificity of visual inspection to detect all grades of dysplasia, atypia and malignancy on cervical cytology were 92.5% and 37.1% respectively (Bharghava et al, 1993). In a study involving 3602 symptomatic and referred women at an early cancer detection centre in Ernakulam, Kerala, the sensitivity and specificity were 92.6% and 37.7% respectively (Sujathan et al, 1995).

The above studies predominantly addressed the feasibility of using paramedical workers to perform speculum examinations and

© Cancer Research Campaign 1997

**Table 6** Results from other studies on the validity of visual inspection

| Reference | Visual inspection findings | Cytology/biopsy findings | | Sensitivity (%) | Specificity (%) |
|---|---|---|---|---|---|
| | | Positive | Negative | | |
| Singh et al (1992) | Positive | 149 | 4986 | 62.6 | 88.9 |
| | Negative | 89 | 39 746 | | |
| Bhargava et al (1993) | Positive | 184 | 2145 | 92.5 | 37.1 |
| | Negative | 15 | 1264 | | |
| Sujathan et al (1995) | Positive | 75 | 2192 | 92.6 | 37.7 |
| | Negative | 6 | 1329 | | |

assessed their capability to identify abnormal cervices and to take Pap smears. The high sensitivity observed in two of the reports is likely to be due to the selected and the symptomatic population studied. A low specificity has been consistently reported. However, the implications of the poor test characteristics were not discussed in these studies.

The present investigation, which involved both symptomatic and asymptomatic individuals, is not a true community-based study. Two thresholds were used to define a positive test to evaluate their effect on the test performance. At a lower threshold of positive visual inspection, the sensitivity was not satisfactory and the test was highly non-specific: the specificity was around 55%. In a setting without facilities for cytology, this would entail recalling 45% of the screened women for examination by gynaecologists. At a high threshold of positive visual inspection, the sensitivity for detecting lesions was even lower.

If one considers cost saving as a major consideration in using low-technology methods in cervical cancer control, unaided visual inspection is unlikely to achieve this objective. If it is used for preselecting women for cytology, the costs saved pertain to not offering Pap smears to women with apparently healthy cervices (approximately 50%). Costs are involved in locating, inviting women for pelvic examination, further referrals and investigations. Results of cost evaluation of cervical cytology programmes in the Netherlands indicate that co-ordination, invitation, pelvic examination and registration account for 55% of costs, and cytological evaluation accounts for 45% (Koopmanschap et al, 1990). As the test is highly unsatisfactory at a high threshold, the lower threshold would entail 45% of the women being recalled, offered Pap smear, and further investigations (colposcopy, biopsy) would be needed in a significant proportion of the women called for Pap smear. At the same time, the low sensitivity would result in at least one third of women with high grade lesions being missed. The need to repeat the visual inspection at periodic intervals would also entail costs. Thus, cost savings are unlikely to be substantial, added to the low efficacy to detect precursor and invasive lesions.

In settings where facilities for even limited cytology do not exist, visual inspection would necessitate half of the women being further examined by gynaecologists, resulting in overcrowding of services.

There might be a concern that some of the false positives on visual inspection are really true positives, misclassified by the results of cytology, and may be responsible for the low specificity and positive predictive value. The Pap smear is admittedly an imperfect screening test with a significant error rate and a wide range of values for sensitivity and specificity in different settings (Shingleton et al, 1995). However, subjects with negative findings on visual inspection may also be misclassified by cytology, and so it is unlikely that sensitivity has been markedly underestimated.

Another concern with visual inspection is the objectivity of the positive test. A screening test should be as objective as possible. Even though defined criteria exist for an abnormal visual finding, the possibility of a subjective decision on the part of the examiners cannot be ruled out. Even after rigorous training, this may result in considerable fluctuations in the validity of unaided visual inspection as a screening test.

It is worthwhile investigating adjuvants to visual inspection. For example, visual examination of 3–5% acetic acid impregnated cervix with the use of a magnifying device (gynoscopy) or without magnification (cervicoscopy) may improve the objectivity and performance of unaided visual inspection. Acetic acid visualization of the cervix (cervicoscopy) has been shown to detect dysplasia, otherwise missed by cervical cytology. A report from Italy (Ceccini et al, 1993) indicated a higher sensitivity of cervicoscopy to detect high-grade lesions than the use of Pap smear, though specificity was lower. Cervicoscopy, therefore, merits evaluation as a screening test.

In summary, the test characteristics of unaided visual inspection are not very promising as a preselection procedure for cytology or as a low technology measure for cervical cancer screening. A reasonable proportion of preinvasive lesions can only be found when large numbers of those examined are classified as 'positive'. Implementation of a public health policy, based on unaided visual inspection for cervical cancer control, is unlikely to be cost-effective and, in the long run, it may prove to be more expensive than a limited cytology programme.

### ACKNOWLEDGEMENTS

The authors gratefully acknowledge the constructive comments by Drs D M Parkin and P Pisani, Unit of Descriptive Epidemiology, International Agency for Research on Cancer, on a draft format of this paper. We are thankful to Mme O Bouvy and Mrs E Bayle, who prepared this manuscript.

### REFERENCES

Bharghava VL, Verma K, Sharma R, Batra S and Anandalakshmy PN (1993) A hospital based study on the use of paramedical personnel for clinical downstaging of cancer cervix. *Ind J Med Res* **98**: 65–68

Cecchini S, Bonardi R, Mazzotta A, Grazzini G, Iossa A and Ciatto S (1993) Testing cervicography and cervicoscopy as screening tests for cervical cancer. *Tumori* **79**: 22–25

Koopmanschap MA, Lubbe KTN, van Oortmarssen GJ, van AGT HMA, van Ballegooijen and Habbema JDF (1990) Economic aspects of cervical cancer screening. *Soc Sci Med* **30**: 1081–1087

© Cancer Research Campaign 1997

Miller AB (1992) *Cervical Cancer Screening Programmes. Managerial Guidelines*. World Health Organization: Geneva

Murthy NS, Agarwal SS, Prabhakar AK, Sharma S and DAS DK (1993) Estimation of reduction in life-time risk of cervical cancer through one life-time screening. *Neoplasma* **40**: 255–258

Parkin DM (1991) Screening for cervix cancer in developing countries. In *Cancer Screening. International Union Against Cancer*. Miller AB, Chamberlain J, Day NE, Hakama M and Prorok PC (eds), pp 184–198, Cambridge University Press: Cambridge

Parkin DM, Pisani P and Ferlay J (1993) Estimates of the worldwide incidence of eighteen major cancers in 1985. *Int J Cancer* **54**: 594–606

Prabhakar AK (1992*a*) Strategy for control of cervical cancer in India. Thesis submitted to the University of Tampere, Tampere, Finland

Prabhakar AK (1992*b*) Cervical cancer in India: strategy for control. *Ind J Cancer* **29**: 104–109

Seghal A, Singh V Bhampani S and Luthra UK (1991) Screening for cervical cancer by direct inspection. *Lancet* **338**: 282

Sherris J, Wells ES, Tsu VD and Bishop A (1993) *Cervical Cancer in Developing Countries: A Situation Analysis*. Women's Health and Nutrition Working paper, The World Bank

Shingleton HM, Patrick RL, Johnson WW and Smith RA (1995) The current status of the Papanicolaou smear. *CA Cancer J Clin* **45**: 305–320

Singh V, Seghal A and Luthra UK (1992) Screening for cervical cancer by direct inspection. *Br Med J* **304**: 534–535

Stjernsward J, Eddy D, Luthra UK and Stanley K (1987) Plotting a new course for cervical cancer screening in developing countries. *World Health Forum* **8**: 42–45

Sujathan K, Kannan S, Pillai KR, Mathew A, Joesph M, Syamalakumari B and Nair MK (1995) Implications of gynaecological abnormalities in pre-selection criteria for cervical cancer screening: preliminary evaluation of 3602 subjects in south India. *Cytopathology* **6**: 75–87

Wilson JMG and Jungner G (1968) *Principles and Practice of Screening for Disease*. Public Health Papers No. 34. World Health Organization: Geneva

World Health Organization (1986) Control of cancer cervix, a WHO meeting. *Bull WHO* **64**: 607–618

© Cancer Research Campaign 1997